# Supplementary material for: Efficiency optimization for large-scale droplet-based electricity generator arrays with integrated microsupercapacitor arrays
Source: Nat Commun. 2025 Sep 26;16:8530. doi: 10.1038/s41467-025-64289-y (PMC12475142; doi:10.1038/s41467-025-64289-y)
Supplement: Supplementary file 1 — Supplementary Information [file 41467_2025_64289_MOESM1_ESM.pdf]

Supplementary Information

Efficiency Optimization for Large-Scale Droplet-Based Electricity Generator Arrays with Integrated Microsupercapacitor Arrays

Zheng Li[1], Shiqian Chen[1], Yujie Fu[1], & Jiantong Li[1*]

[1]KTH Royal Institute of Technology, School of Electrical Engineering and Computer Science, Division of Electronics and Embedded Systems, Stockholm, Sweden.

[*]Corresponding author: Jiantong Li, jiantong@kth.se

Table of Contents:

Supplementary Note 1

Supplementary Figures 1-28

Supplementary Tables 1-3

Supplementary Videos 1-5

Supplementary References

Supplementary Note 1

Derivation of Equation (1)

Equation (1) is derived according to the same procedure in the previous research[1]. The circuit model is simplified as shown in Figure 2a where the electric double layer (EDL) capacitors between the droplet and top electrode and between the droplet and PTFE film are neglected. When a droplet spreads on the PTFE surface, mechanical energy is converted into electrical energy and stored in C_B with the stored charge $Q = C_B U_0$, where U_0 is the intrinsic initial voltage across C_B. Subsequently, the charge is transferred to the external circuit (stored in the parasitic circuit capacitance $C_{P,C}$ and the parasitic device capacitor $C_{P,D}$ between the top and bottom electrodes. As $C_{P,C}$ and $C_{P,D}$ are in parallel, the total parasitic capacitance is $C_P = C_{P,C} + C_{P,D}$. During the charge transfer, the voltage across C_B reduces from U_0 to U_B, while the voltage across C_P increases from 0 to U_P. At equilibrium, $U_B = U_P$. The transferred charge Q_T is therefore

$$Q_T = C_P \cdot U_P = C_B \cdot (U_0 - U_B) = C_B \cdot (U_0 - U_P). \qquad \text{(S1)}$$

Because the EDL capacitors are neglected, the peak voltage obtained in the measuring circuit is equal to U_P at equilibrium. From Equation (S1), one obtains

$$V_{peak} = U_P = \frac{U_0 C_B}{C_B + C_P} = \frac{U_0 C_B}{C_B + C_{P,D} + C_{P,C}}. \qquad \text{(S2)}$$

Measurement of $C_{P,C}$ under different S_{BE}

The parasitic circuit capacitance $C_{P,C}$ is measured according to the protocol in the previous research[23]. As illustrated in Figure S8a, an external variable capacitor C_E is connected between the top and bottom electrodes of the DEGs so that C_E is parallel with C_P. One may then replace C_P in Equation (S2) with $C'_P = C_P + C_E$ and obtain the C_E-dependent peak voltage V_{peak} as

$$V_{peak} = \frac{C_B U_0}{C_B + C'_P} = \frac{Q}{C_B + C_{P,D} + C_{P,C} + C_E} = \frac{\sigma_S S_{eff}}{C_B + C_{P,D} + C_{P,C} + C_E}. \qquad \text{(S3)}$$

For a DEG with specified structure, C_B and $C_{P,D}$ are fixed while $Q = C_B U_0 = \sigma_S S_{eff}$ is also fixed with σ_S being the permanent surface charge density on the PTFE film and $S_{eff} = \min(S_{BE}, S_{D,max})$ being the minimal value between the BE area and the maximum droplet spread area. Then one can measure a series of V_{peak} with varying C_E and extract the value of $C_{P,C}$ from Equation (S3). In this work, for each DEG with S_{BE} ranging from 1 cm^2 to 180 cm^2,

we vary C_E from 0 to 1000 pF to measure V_{peak}. Equation (S3) is used to fit the experimental data with σ_S = 48 μC m^{-2} and extract the values of $C_{P,C}$ (Figure 2e). The surface charge of density of PTFE film σ_S = 48 μC m^{-2} used in our fitting is comparable to the value of ~ 35 μC m^{-2} in the previous research[1].

Supplementary Figures

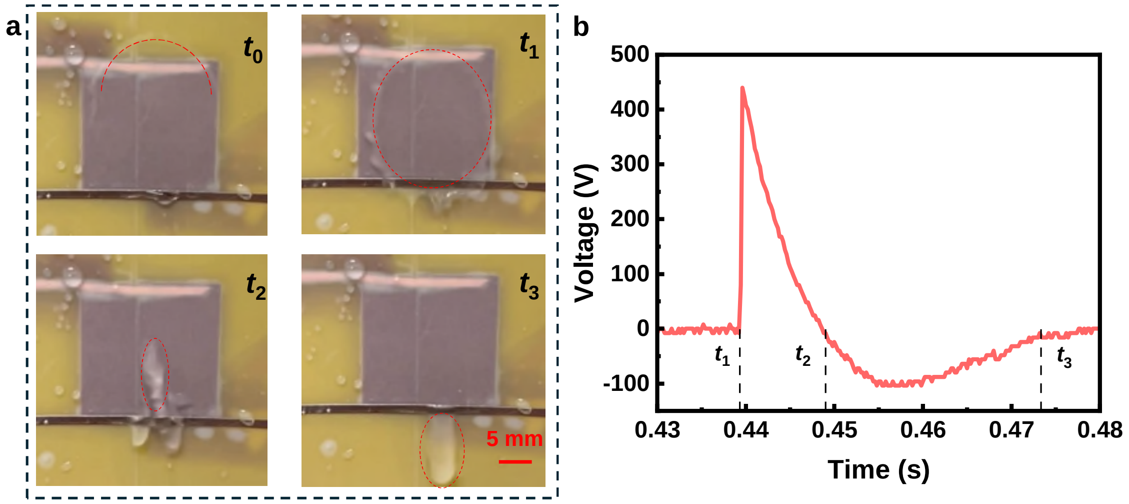

Supplementary Figure 1| Dynamics of a water droplet. a Spreading process of a water droplet on the PTFE surface. The droplet starts to touch the PTFE surface at the time of t_0, spreads to the maximum area and touches the top electrode at t_1, recedes during period t_1 - t_2 - t_3, and finally detaches from the top electrode at t_3. **b** Typical time-resolved voltage curve of a DEG cell for one droplet impinging. Source data are provided as a Source Data file.

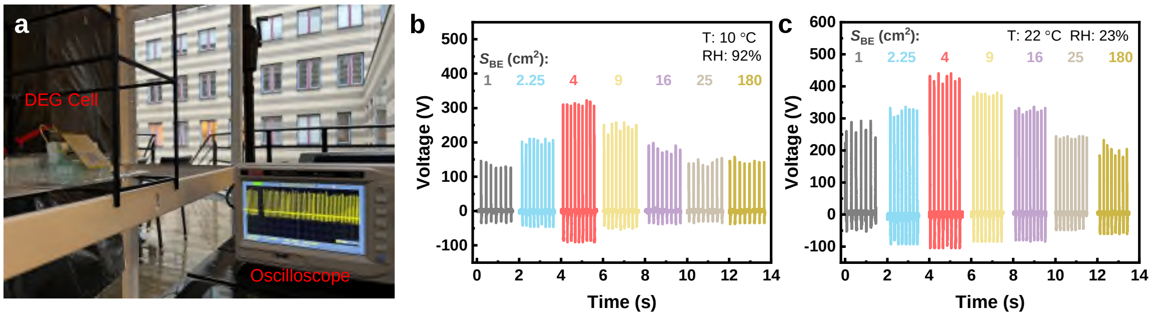

Supplementary Figure 2| Outdoor test of DEG cells with different bottom electrode area S_{BE} driven by DI water. a Photograph of one DEG cell operated outdoors (Temperature: 10 °C, Relative Humidity: 92%). **b,c** Output voltage of the DEG cells tested under (b) outdoor and (c) indoor environments. Source data are provided as a Source Data file.

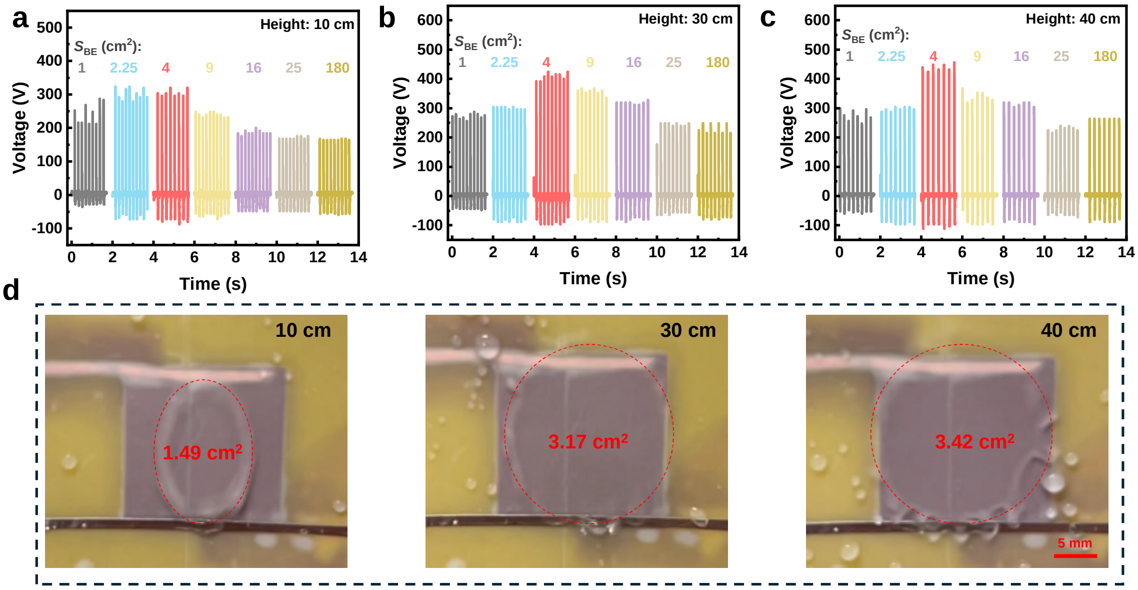

Supplementary Figure 3| Output performance of DEG cells of various bottom electrode area S_{BE} driven by droplets from different falling heights. a-c Output voltage of the DEG cells with droplet falling heights of (a) 10 cm, (b) 30 cm, and (c) 40 cm. **d** Photographs of the droplets with different falling heights just spreading to the maximum area on the PTFE film. Source data are provided as a Source Data file.

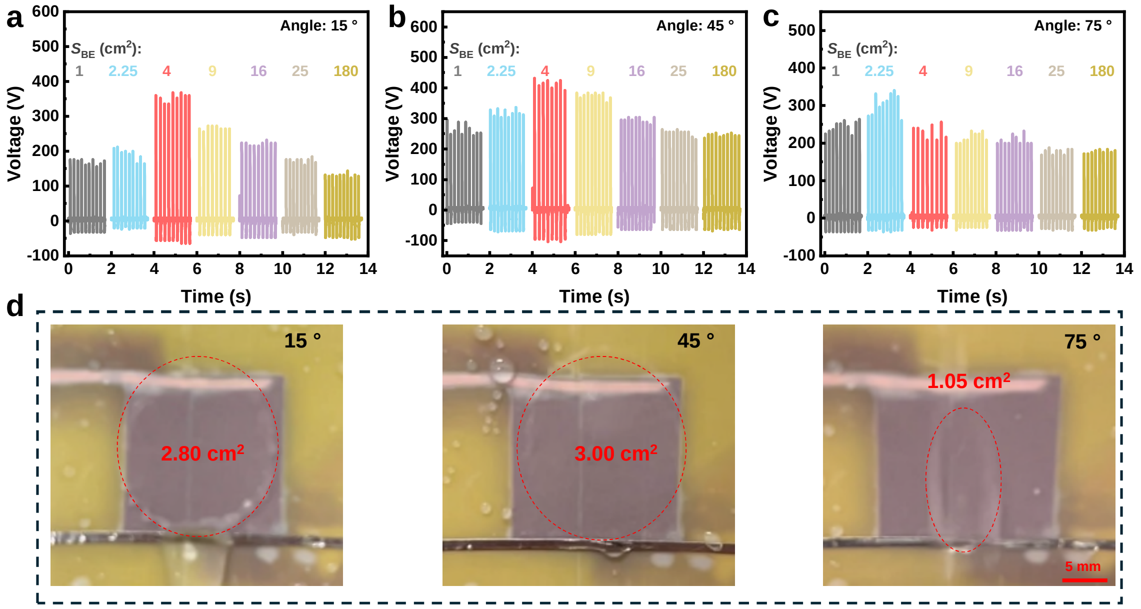

Supplementary Figure 4| Output performance of DEG cells of various bottom electrode area S_{BE} driven by droplets with different impact angles. a-c Output voltage of the DEG cells with droplet impact angles of (a) 15°, (b) 45°, and (c) 75°. **d** Photographs of the droplets with different impact angles just spreading to the maximum area on the PTFE film. Source data are provided as a Source Data file.

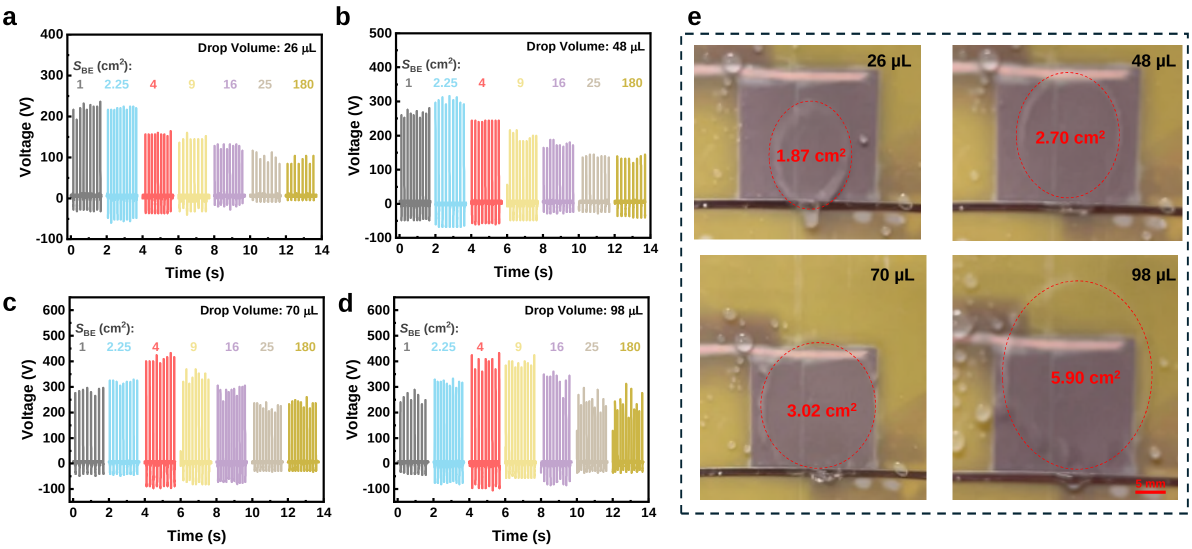

Supplementary Figure 5| Output performance of DEG cells of various bottom electrode area S_{BE} driven by droplets of different volumes. a-d Output voltage of the DEG cells with the droplet volumes of (a) 26 μL, (b) 48 μL, (c) 70 μL, and (d) 98 μL. The droplet volume is controlled by the inner diameter of the nozzles in the droplet generators. In (a-d), the inner nozzle diameters are 1.8 mm, 2.2 mm, 3.4 mm, and 4.5 mm, respectively. **e** Photographs of the droplets with different volumes just spreading to the maximum area on the PTFE film. Source data are provided as a Source Data file.

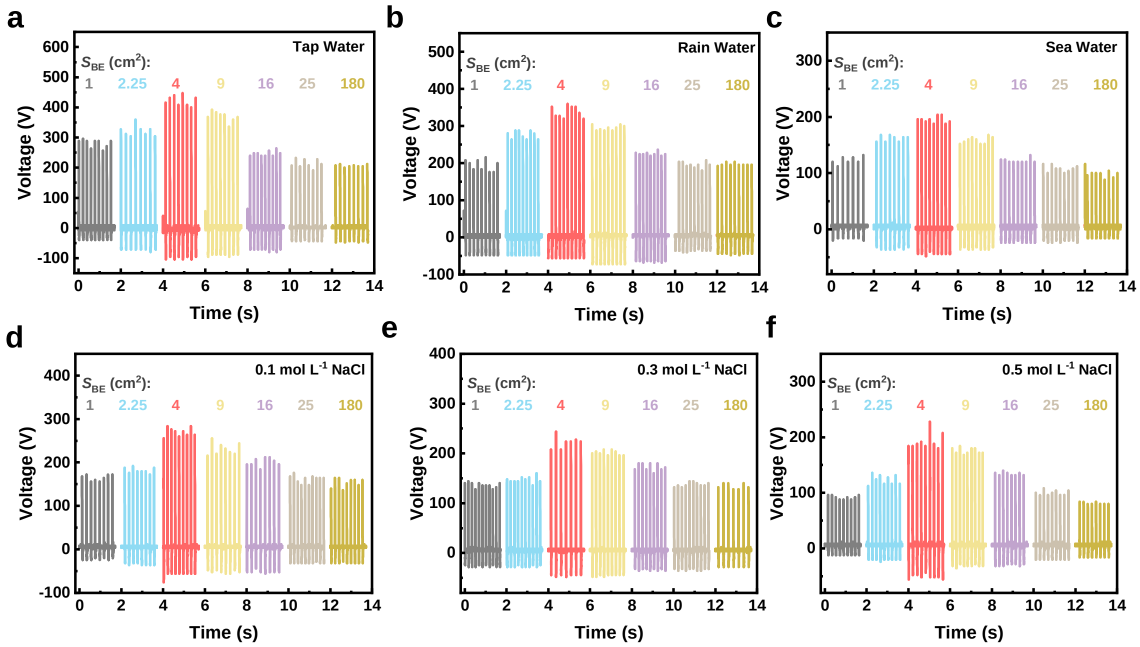

Supplementary Figure 6| Output performance of DEG cells of various bottom electrode area S_{BE} driven by different types of water. a-c Output voltage of the DEG cells driven by (a) tap water, (b) rainwater, and (c) seawater. **d-f** Output voltage of the DEG cells driven by aqueous NaCl solutions of different concentrations: (d) 0.1 mol L^{-1}, (e) 0.3 mol L^{-1}, and (f) 0.5 mol L^{-1}. Source data are provided as a Source Data file.

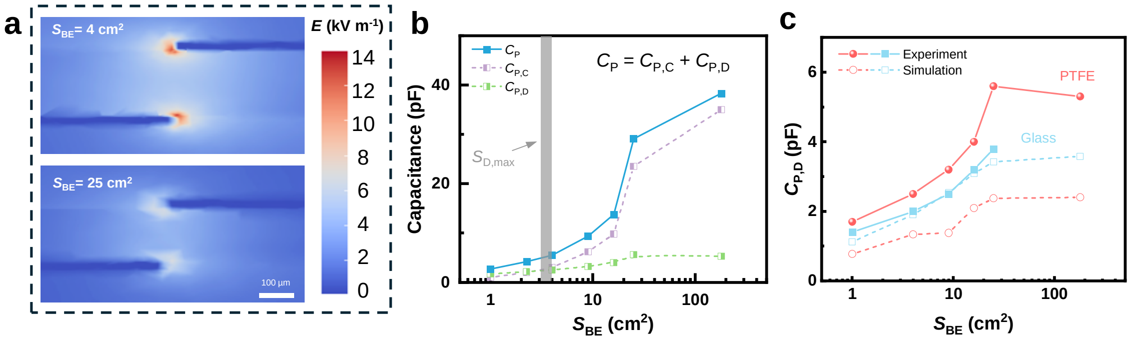

Supplementary Figure 7| Simulation and experimental measurement of parasitic capacitance. a FEM simulated electric field distribution on cross-section of the DEG cells with different S_{BE}. **b** Experimentally measured dependence of the total parasitic capacitance C_P on S_{BE} for PTFE dielectric film. **c** Dependence of $C_{P,D}$ on S_{BE} for PTFE and glass dielectric film from both experimental measurement and simulation results. Source data are provided as a Source Data file.

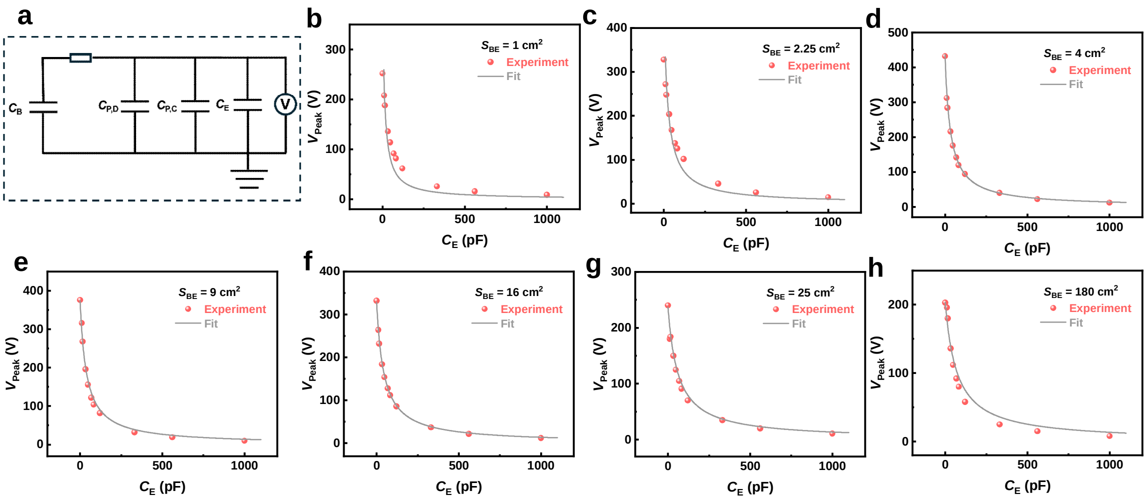

Supplementary Figure 8| Measurement of parasitic circuit capacitance $C_{P,C}$. a Equivalent electric circuit of the DEG cell with an external load capacitor C_E. **b-h** Peak voltage dependence on C_E for the DEG cell with different S_{BE}. Source data are provided as a Source Data file.

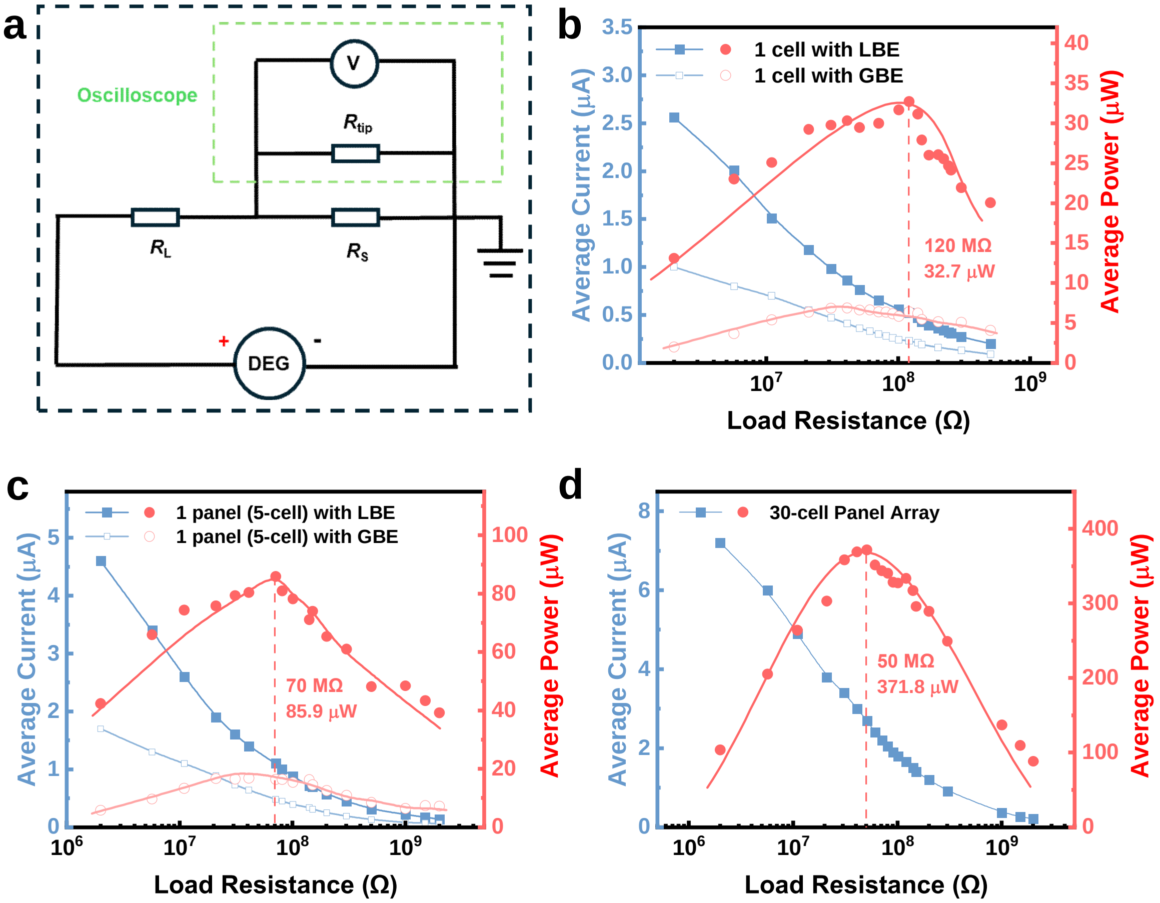

Supplementary Figure 9| Average current and power against load resistance for various DEG devices. a Equivalent circuit for measuring the output current of a DEG with load resistance R_L through the voltage divider method. In all our tests, R_S = 1 MΩ and R_{tip} = 100 MΩ. **b-d** Average current and power of the (b) DEG cell, (c) panel, and (d) panel arrays. The average voltage U_{RMS} is calculated according to Equation (2). The average current is $I_{RMS} = U_{RMS}/R_s$ and average power $P_{RMS} = I_{RMS}^2(R_S + R_L)$. Source data are provided as a Source Data file.

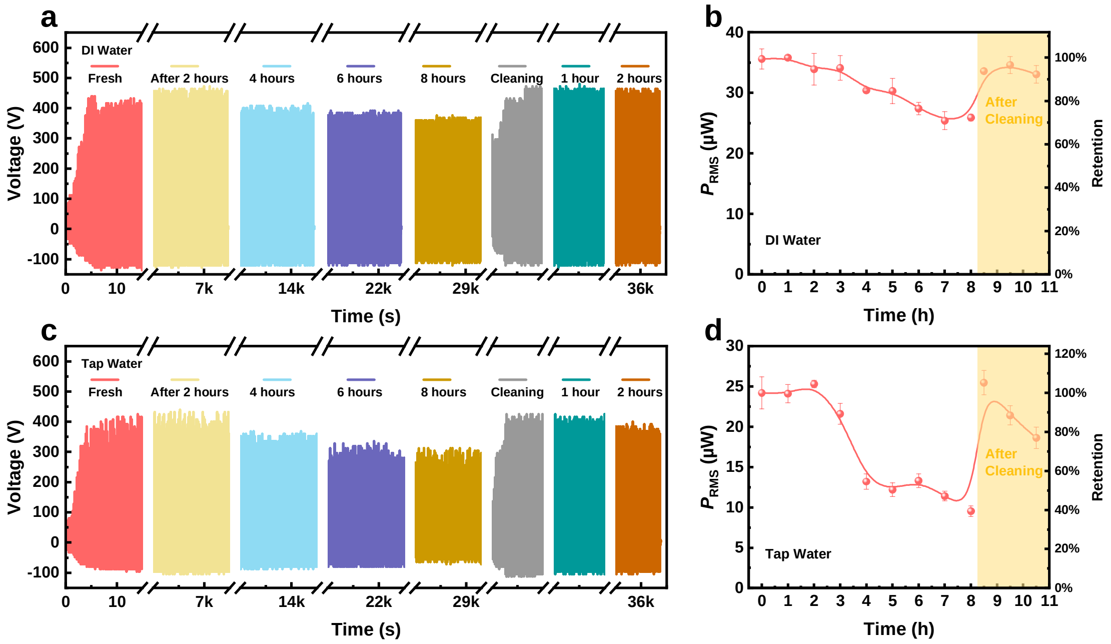

Supplementary Figure 10| Long-term stability test of a DEG cell. a,c Output voltage-time curves of the DEG cell driven by (a) DI water and (c) tap water. In each case, after a test time of 8 hours, the DEG cell was cleaned though wiping the PTFE surface with paper tissue. **b,d** Time-resolved average power P_{RMS} and power retention of the DEG cell driven by (b) DI water and (d) tap water. Error bars represent the standard sample deviation. Source data are provided as a Source Data file.

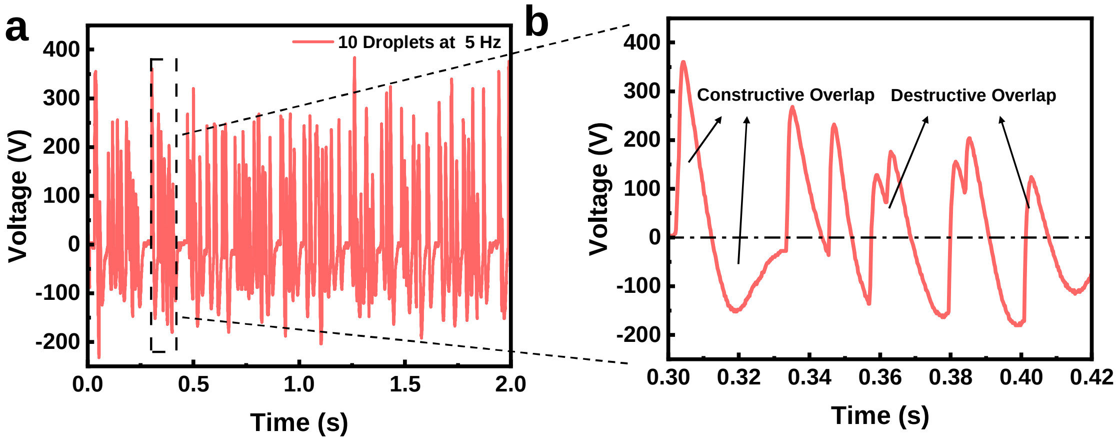

Supplementary Figure 11| Electrical interference of the DEG panel. a Output voltage of a 10-cell DEG panel. **b** Enlarged view of the voltage signal. Source data are provided as a Source Data file.

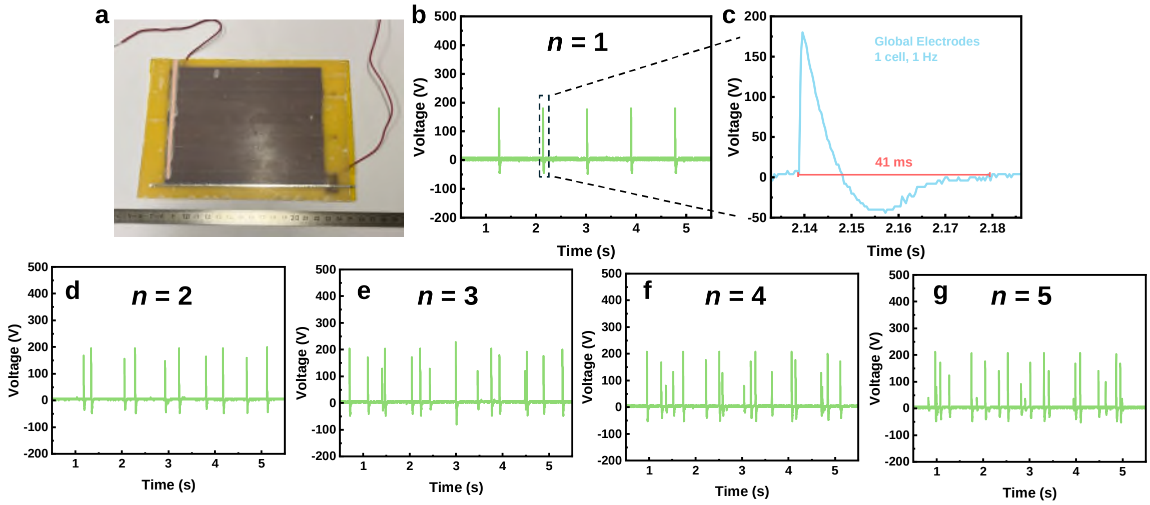

Supplementary Figure 12| Performance of the GBE panel with different cell numbers at a droplet frequency of 1 Hz. a Photograph of the DEG panel with GBE structure. **b** Output voltage of 1-cell GBE panel. **c** Closeup view of the voltage-time curve for one impinging droplet. **d-g** Output voltage of the GBE panel with different number (from 2 to 5) of working DEG cells. Source data are provided as a Source Data file.

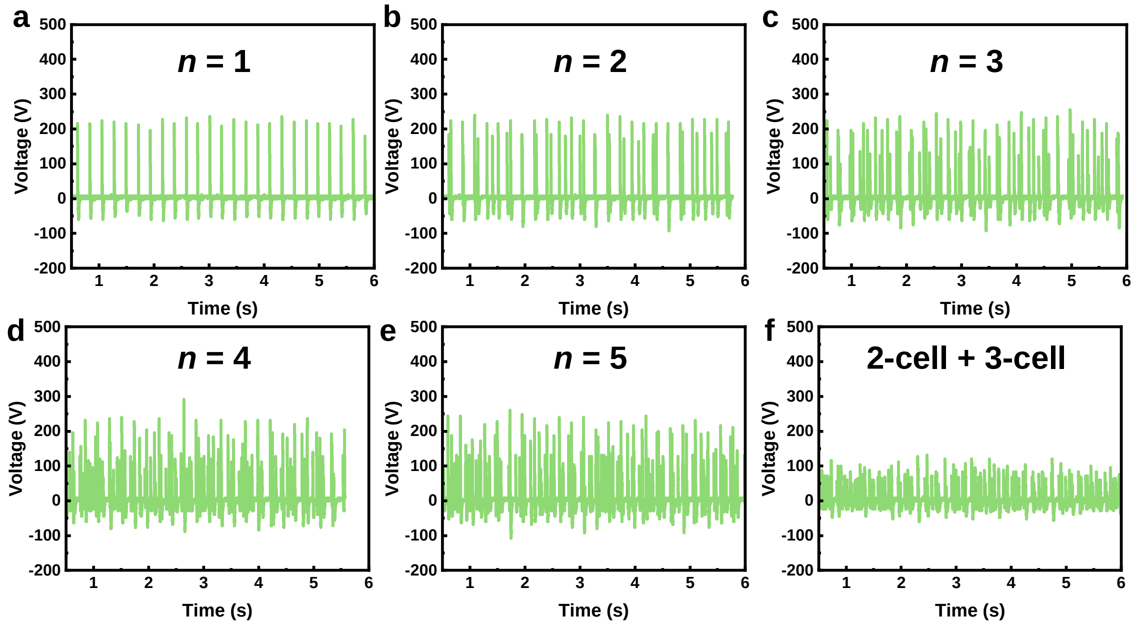

Supplementary Figure 13| Performance of the GBE panel with different cell numbers at a droplet frequency of 5 Hz. a-e Output voltage of the GBE panel with different cell numbers (from 1 to 5). **f** Output voltage of two connected (2-cell and 3-cell) GBE panels. Source data are provided as a Source Data file.

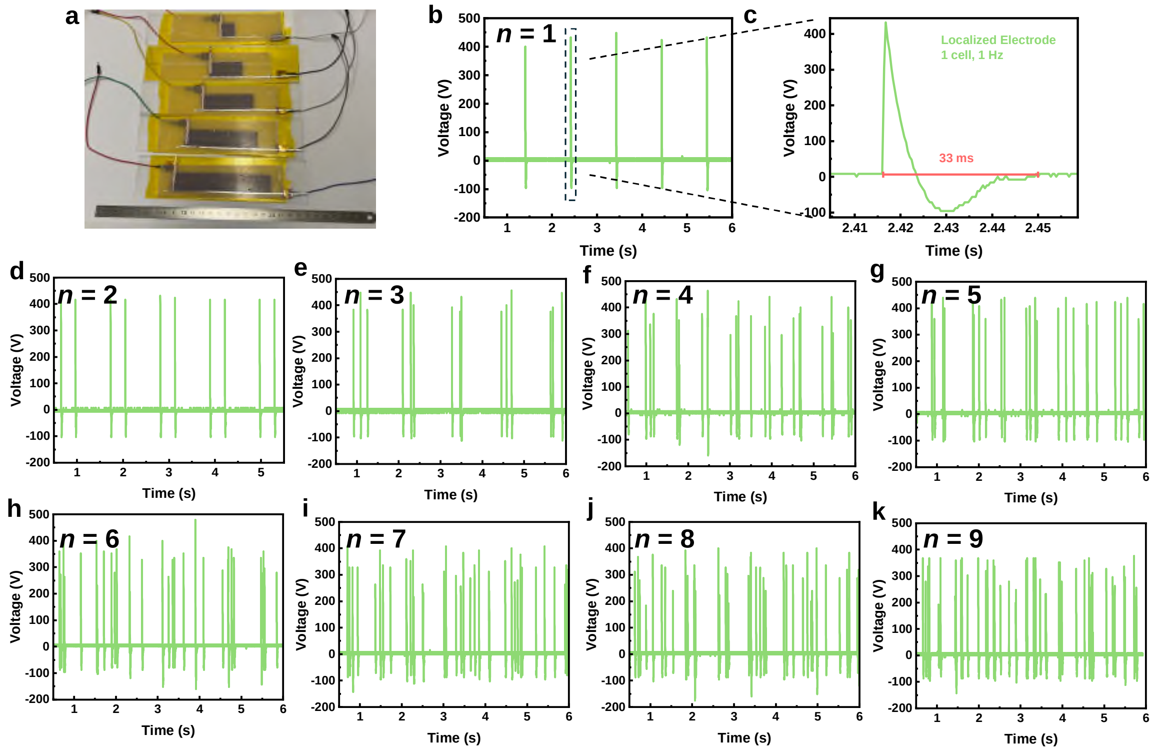

Supplementary Figure 14| Performance of the LBE panels with different cell numbers at a droplet frequency of 1 Hz. a Photograph of the LBE panels for different cell numbers. **b** Output voltage of 1-cell LBE panel. **c** Close-up view of voltage-time curve for one water droplet. **d-k** Output voltage of the LBE panels with different cell numbers (from 2 to 9). Source data are provided as a Source Data file.

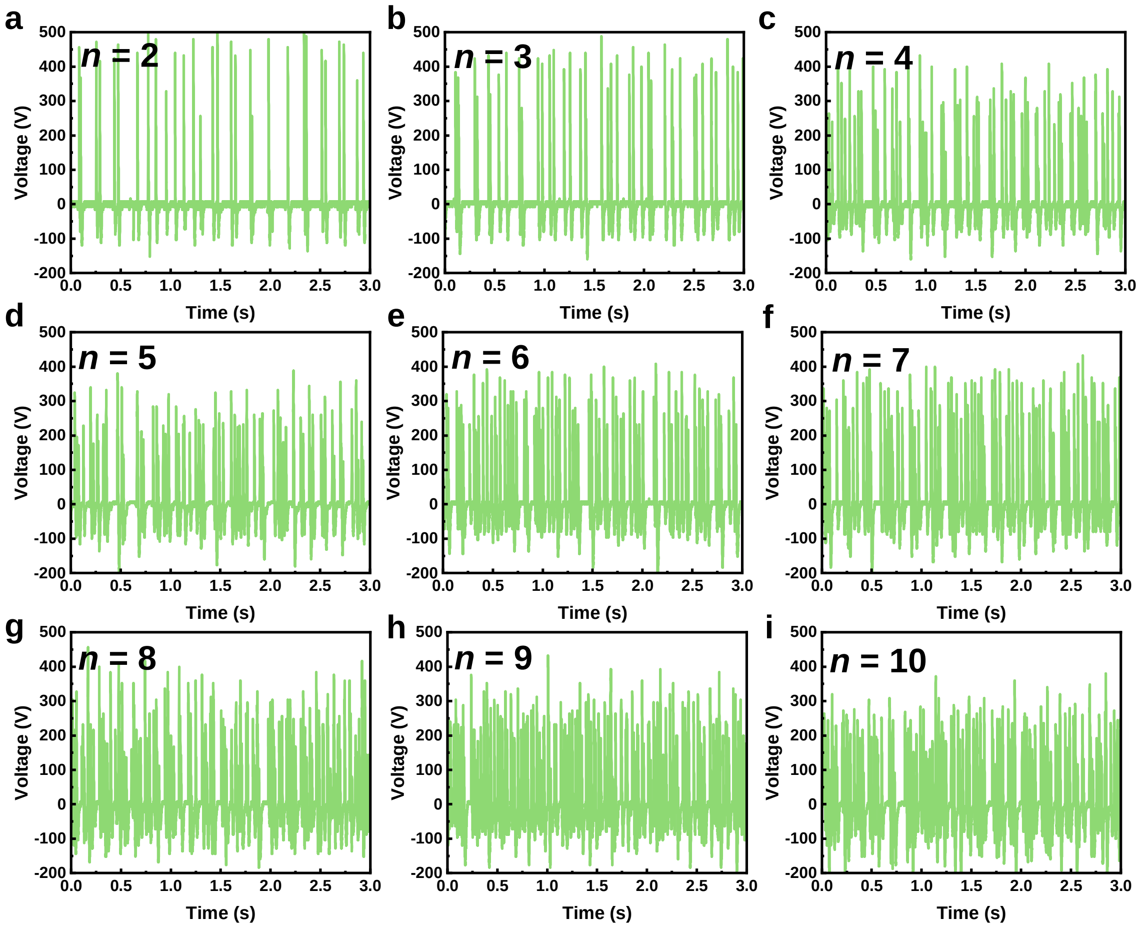

Supplementary Figure 15| Output voltage of the LBE panels with different cell numbers at a droplet frequency of 5 Hz. a-i Output voltage of LBE panel with different cell numbers (from 2 to 10). Source data are provided as a Source Data file.

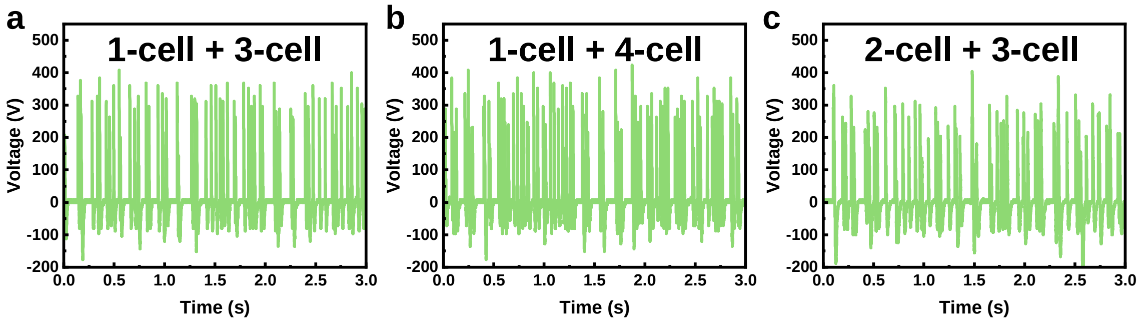

Supplementary Figure 16| Output voltage of two connected LBE panels with different cell numbers at a droplet frequency of 5 Hz. a Connection between a 1-cell panel and a 3-cell panel. **b** Connection between a 1-cell panel and a 4-cell panel. **c** Connection between a 2-cell panel and a 3-cell panel. Source data are provided as a Source Data file.

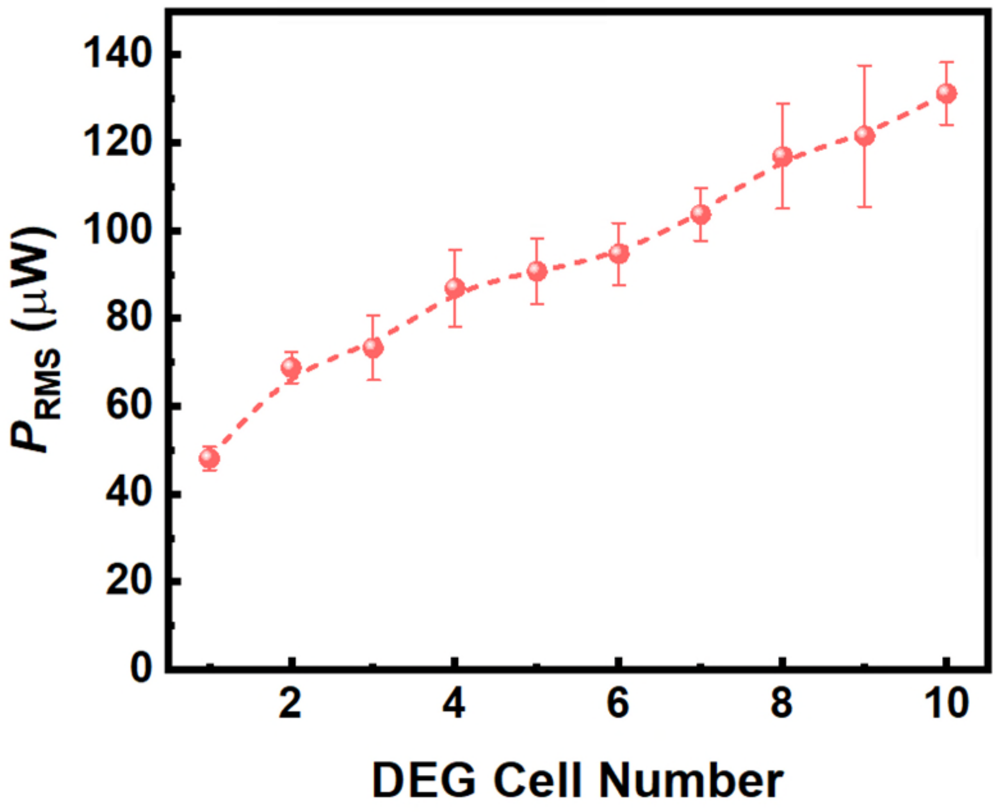

Supplementary Figure 17| Average power of the LBE panel with different cell numbers at a droplet frequency of 8 Hz. Error bars represent the standard sample deviation. Source data are provided as a Source Data file.

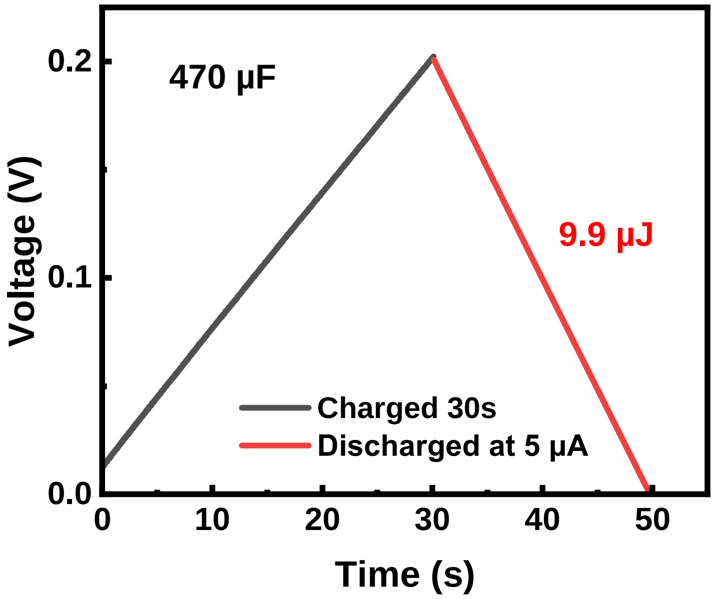

Supplementary Figure 18| Voltage-time curve of a commercial capacitor (470 μF) charged by the 30-cell DEG panel array for 30 s and then discharged under a current of 5 μA. Source data are provided as a Source Data file.

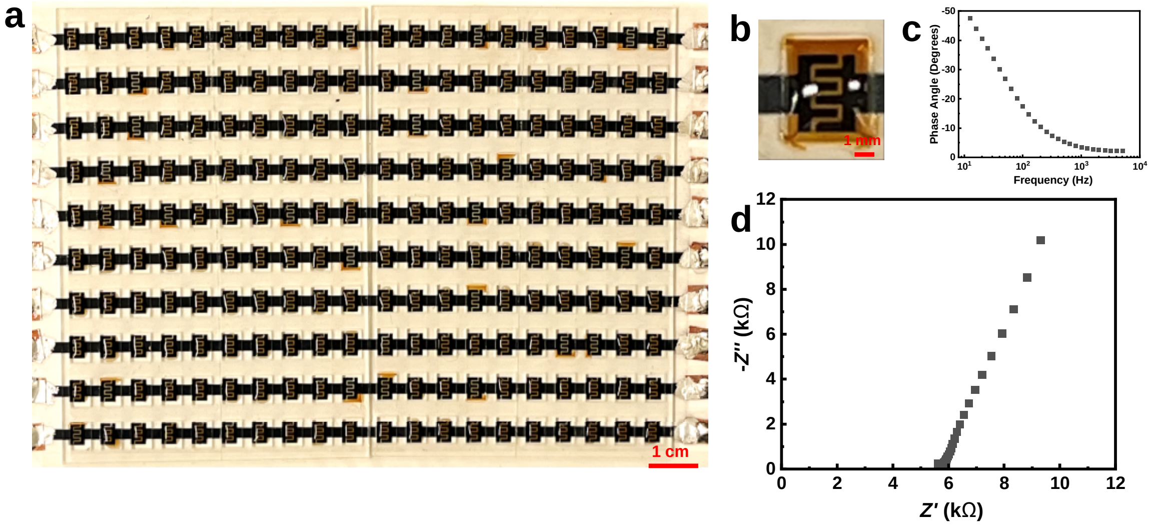

Supplementary Figure 19| Morphology and Electrochemical Characterization of MSC arrays. a Photograph of a 200-cell MSC array with electrolytes. **b** Photograph of a single MSC cell with electrolyte. **c** Bode plot and **d** Nyquist plot of a 100-cell MSC sub-array. Source data are provided as a Source Data file.

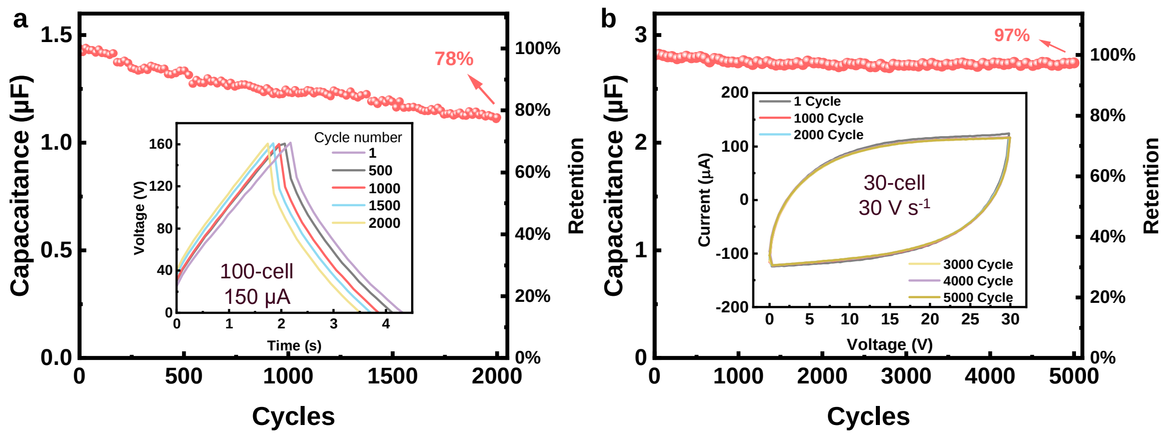

Supplementary Figure 20| Long-cycle charge/discharge tests of MSC arrays. a Cycling test of a 100-cell MSC array for 2000 GCD cycles at a current of 150 μA within a voltage window of 160 V. **b** Cycling test of a 30-cell array for 5000 CV cycles at a scan rate of 30 V s^{-1} within a voltage window of 30 V. Source data are provided as a Source Data file.

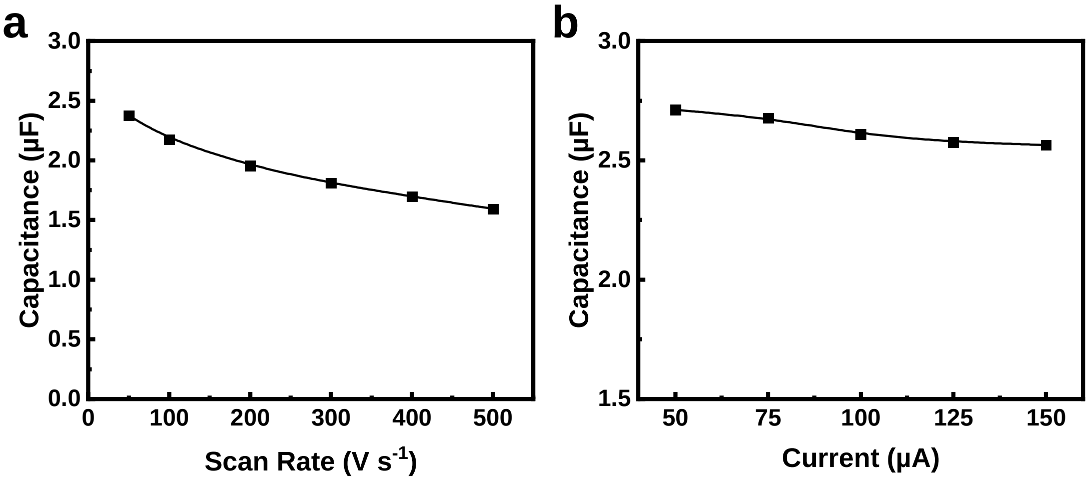

Supplementary Figure 21| Overall capacitance of the 100-cell MSC sub-array in Figure 5. a Capacitance against scan rate. **b** Capacitance against charge-discharge current. Source data are provided as a Source Data file.

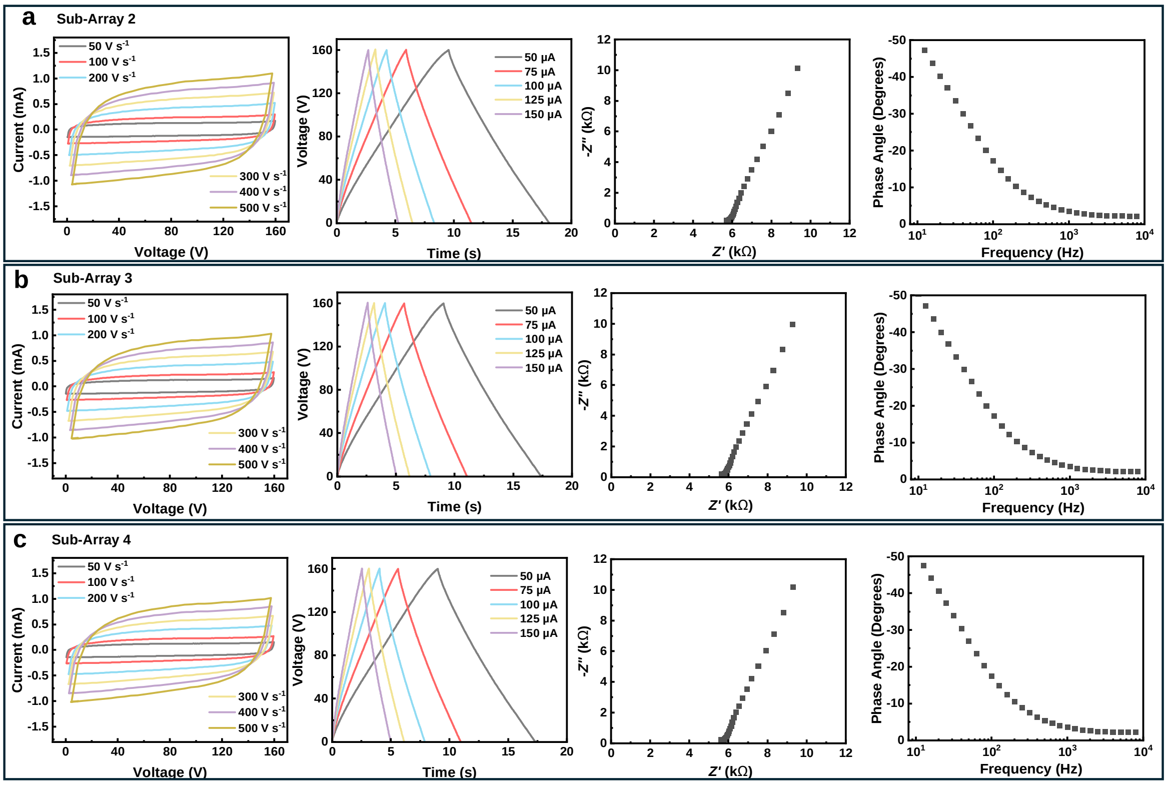

Supplementary Figure 22| Electrochemical Characterization of the other three 100-cell MSC sub-arrays. a-c (from left to right) CV curves at different scan rates, GCD curves under different currents, Nyquist plots and Bode plots for (a) Sub-Array 2, (b) Sub-Array 3 and (c) Sub-Array 4. Source data are provided as a Source Data file.

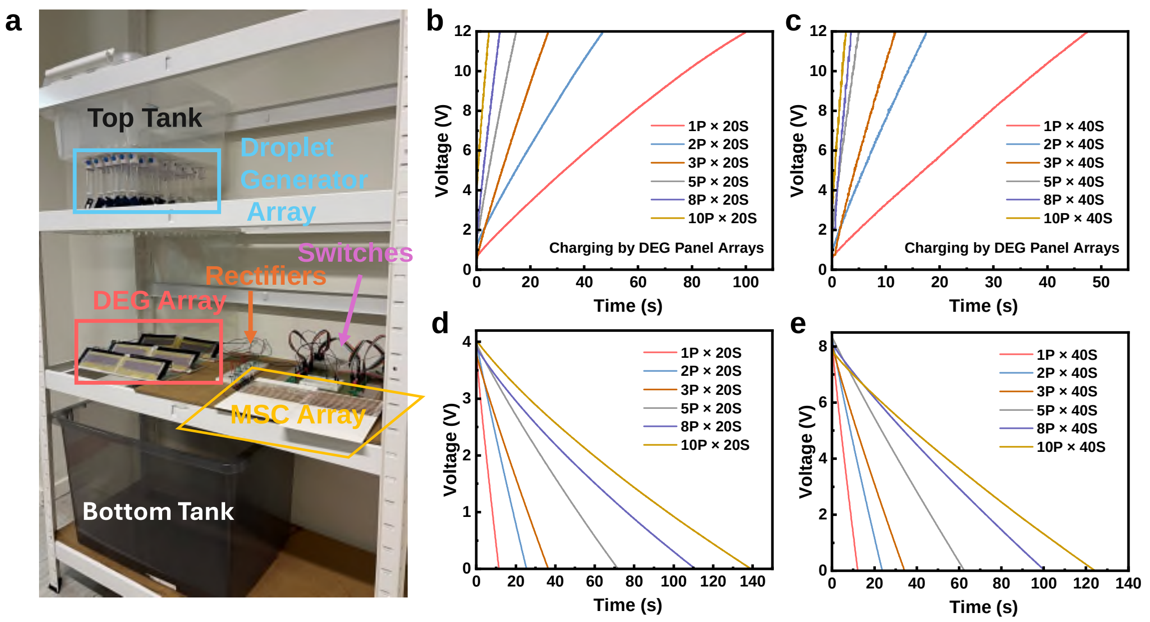

Supplementary Figure 23| Integration and application of the SCPS. a Photograph of the SCPS. **b-c** Charge curves of the MSC arrays with different cell numbers, charged by the 30-cell DEG panel array. **d-e** Discharge curves of the MSC arrays under a current of 5 μA, after they have been charged by the 30-cell DGE panel array for 30 s. Source data are provided as a Source Data file.

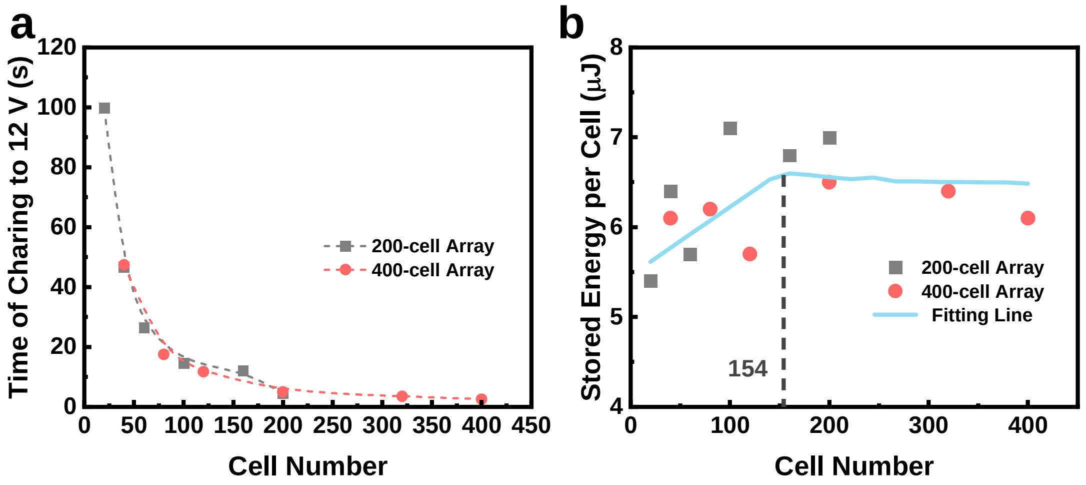

Supplementary Figure 24| Performance dependence on the cell number for the MSC arrays charged by the 30-cell DEG panel array. a Dependence of the time for MSC arrays to be charged to 12 V on the MSC cell number. **b** Dependence of stored energy per cell on the cell number in the MSC arrays after being charged by the DEG panel array for 30 s. Note to investigate the effects of the cell number, in some tests only a part of the MSC cells in the 200-cell and 400-cell arrays are used. Source data are provided as a Source Data file.

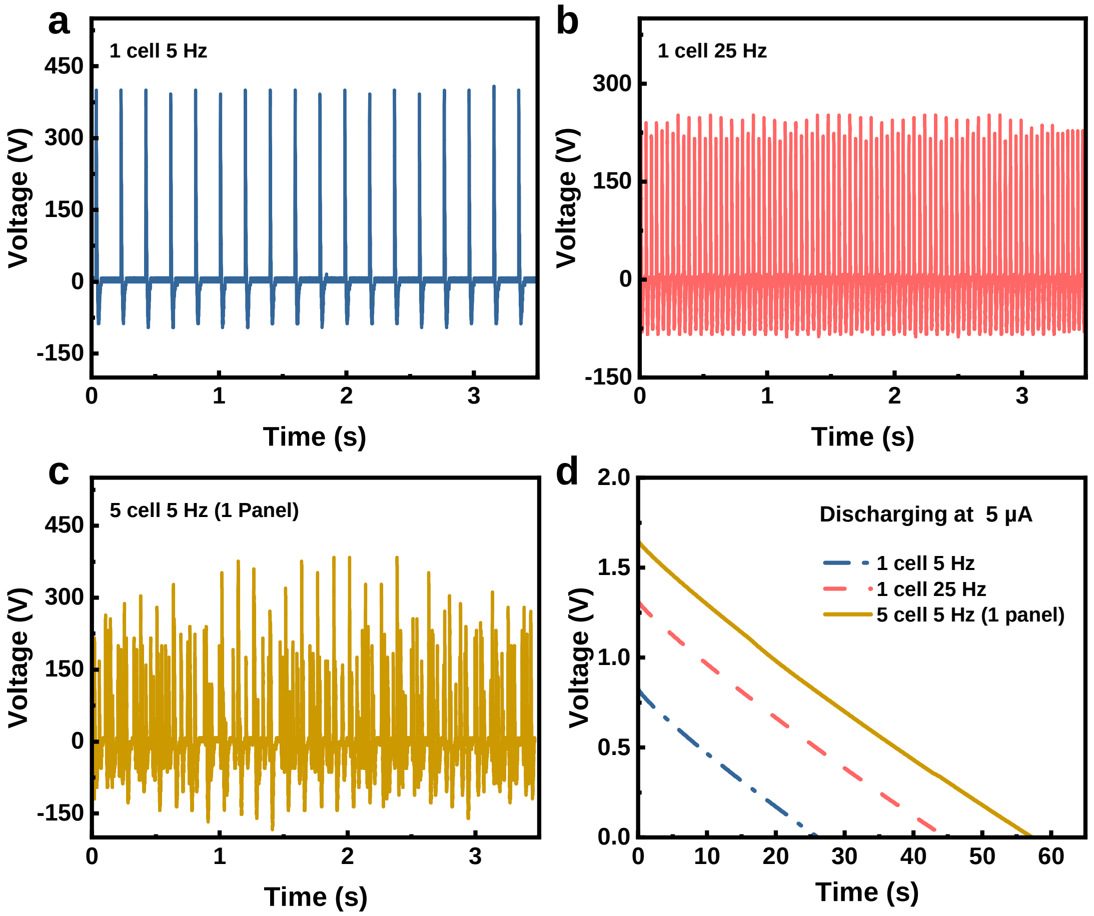

Supplementary Figure 25| Performance of a 200-cell MSC array charged with different DEGs under different operating conditions. a-c Output voltage of the different DEGs (a) one DEG cell at 5 Hz, (b) one DEG cell at 25 Hz, and (c) one DEG panel consisting of 5 DEG cells at 5 Hz. **d** Discharge curves of the 200-cell MSC arrays at a configuration of 10P × 20S under a current of 5 μA, after being charged for 30 s by the three different DEGs. Source data are provided as a Source Data file.

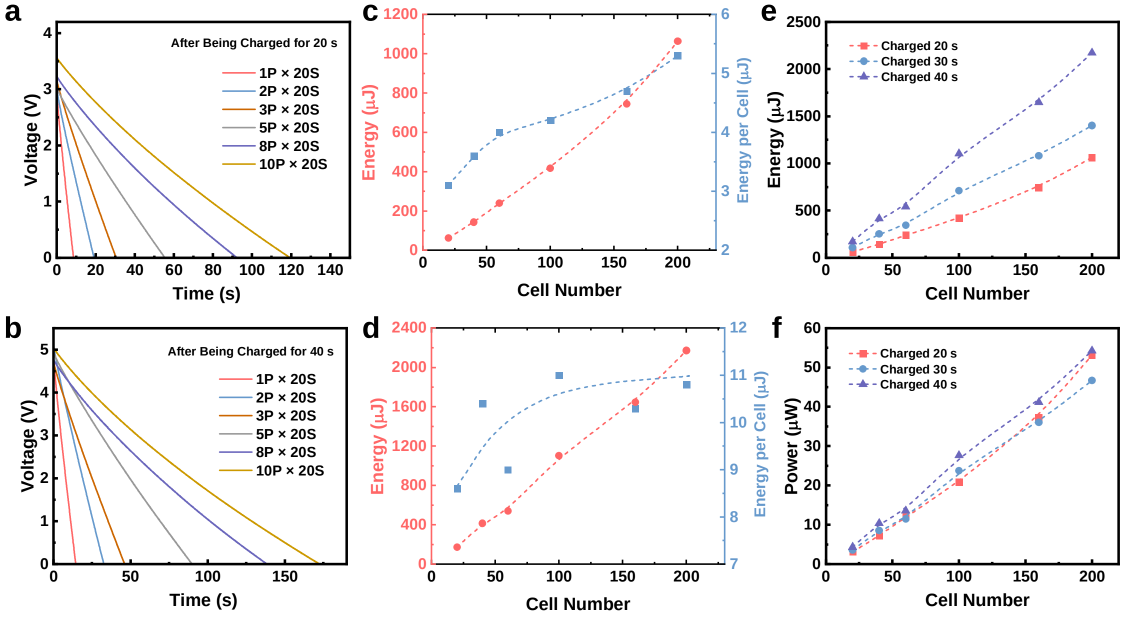

Supplementary Figure 26| Additional Charge/Discharge tests of the MSC arrays with different MSC cell numbers in the SCPS. a,b Discharge curves of the MSC arrays with different cell numbers under a current of 5 μA, after being charged by the 30-cell DEG panel array for (a) 20 s and (b) 40 s. **c,d** Dependence of the total stored energy and stored energy per cell on the cell number for the MSC arrays. **e,f** Stored energy (e) and storing power (f) of the MSC arrays as a function of the cell number after being charged by the DEG panel array for different times. Source data are provided as a Source Data file.

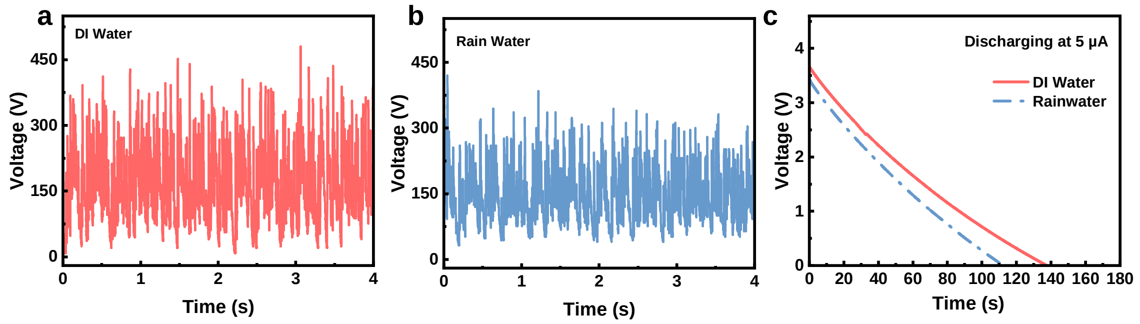

Supplementary Figure 27| Test of charging a 200-cell MSC array by a 30-cell DEG panel array driven by DI water and rainwater. a,b Output voltage of the 30-cell DEG panel array driven by (a) DI water and (b) rainwater. **c** Discharge curves of the 200-cell MSC array at a configuration of 10P × 20S under a constant current of 5 μA, after the MSC array has been charged for 30 s by the DEG panel array with different types of water. Source data are provided as a Source Data file.

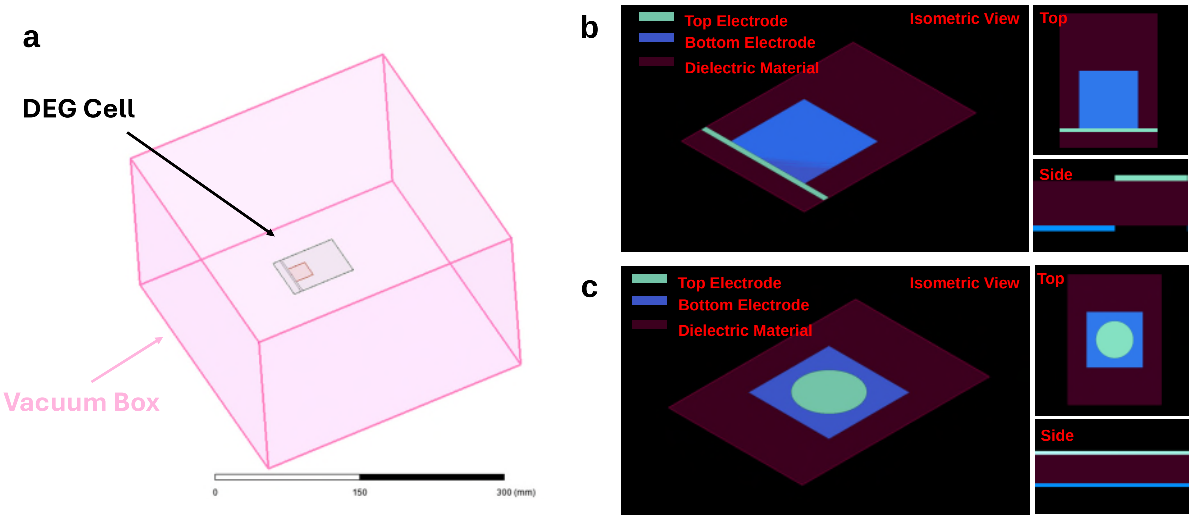

Supplementary Figure 28| Simulation model for capacitance calculation. **a** 3D view of the overall simulation model. The DEG cell is placed at the center of a large vacuum box. **b**,**c** Different (isometric, top and side) views for the simulation models of the DEG cells for calculating (b) $C_{P,D}$ and (c) C_B.

Supplementary Table 1. Comparison of performance among various DEG devices.

Devices	Water Type	Droplet Volume (μL)	Droplet Frequency	Impact Angle (°)	Droplet Height (cm)	Droplet Spreading Area (cm^2)	U_{RMS} (V)	P_{RMS} (μW)	E_V (μJ mL^{-1})	$\hat{P}_{RMS}$ (mW m^{-2}) [a]	ESE	Energy Harvesting Power (μW)	Ref
Cell	Tap Water	100	4.2 Hz	15	15	2.7	11.6	13.4 [b]	31.9	49.4	-	-	[2]
Cell	Tap Water	60	1.5 Hz	45	25	2.6	13.5	6.0 [b]	66.7	27.5	-	-	[1]
Cell	Tap Water	4	165.0 Hz	45	-	0.17	14.6	21.4 [b]	32.4	1256.6	7.5%	1.6	[3]
Cell	DI Water	50	9.0 Hz	35	10	-	25.8	8.7	14.9	8.7	62%	5.4	[4]
Cell	Tap Water	53	-	45	20	2.5	-	-	-	-	-	-	[5]
Cell	Tap Water	72	30 mL min^{-1} (6.9 Hz)	30	16	2.2	-	-	-	-	-	0.4	[6]
Cell	DI Water	70	5 Hz	45	30	3.0	54.8	32.7	93.4	109.0	-	-	This Work
Panel	Tap Water	53	-	45	20	-	-	-	-	-	-	0.8	[5]
Panel	Tap Water	99.5	5.1 Hz	-	15	-	-	-	-	-	-	0.3	[7]
Panel	Tap Water	72	3 Droplet at 50 mL min^{-1} (11.6 Hz)	30	30	2.2	6.3	30.6	12.2	46.4	0.6%	0.2	[6]
Panel	DI Water	70	5 Droplet at 5 Hz	45	30	3.0	86.0	85.9	49.1	57.3	-	-	This Work
Panel Arrays	Tap Water	72	30 Droplets at 20-30 mL min^{-1} (6.9 Hz)	30	30	2.2	50.5	152.2	10.2	23.1	2.0%	3.0	[6]
Panel Arrays	DI Water	70	30 Droplet at 5 Hz	45	30	3.0	177.3	371.8	35.4	41.3	21.8%	81.2	This Work

[a] Average power density, [b] Results are calculated from the product of the generated electrical energy per droplet with the impinging frequency, reported in papers.

Supplementary Table 2. Relevant performance of the SCPSs in Figure S25 to charge a 200-cell MSC array with different DEGs for ***t* = 30 s** under different operating conditions.

DEG	Average Output Power of DEG P_{RMS} (μW)	Harvested Energy per unit droplet volume of DEG E_V (μJ mL^{-1})	Stored Energy in MSC array E_{store} (μJ)	Energy Harvesting Power E_{store}/t (μW)	ESE (%) $E_{store}/(P_{RMS}\ t)$
1-cell at 5 Hz	28	80	52.0	1.7	6.2
1-cell at 25 Hz	42	34	145.1	4.8	11.5
5-cell at 5 Hz	74	47	234.9	7.8	10.6

Supplementary Table 3. Composition of the lab-prepared raindrop-mimicking liquid.

Component	Concentration (mg L^{-1})
NaCl	5
KCl	2
$MgCl_2$	1
$(NH_4)_2SO_4$	3
$CaSO_4$	2
H_2SO_4	Until pH = 5~6

Supplementary References

1. Li, L. et al. Sparking potential over 1200 V by a falling water droplet. *Sci Adv* **9**, eadi2993 (2023).
2. Xu, W. et al. A droplet-based electricity generator with high instantaneous power density. *Nature* **578**, 392-396 (2020).
3. Wang, L.L. et al. Harvesting energy from high-frequency impinging water droplets by a droplet-based electricity generator. *Ecomat* **3**, e12116 (2021).
4. Chen, S. et al. Ultrafast Metal-Free Microsupercapacitor Arrays Directly Store Instantaneous High-Voltage Electricity from Mechanical Energy Harvesters. *Adv Sci (Weinh)* **11**, e2400697 (2024).
5. Xu, X.T. et al. Droplet energy harvesting panel. *Energy & Environmental Science* **15**, 2916-2926 (2022).
6. Ye, C. et al. An Integrated Solar Panel with a Triboelectric Nanogenerator Array for Synergistic Harvesting of Raindrop and Solar Energy. *Adv Mater* **35**, e2209713 (2023).
7. Wang, K.Q. et al. Enhancing water droplet-based electricity generator by harnessing multiple-dielectric layers structure. *Nano Energy* **111**, 108388 (2023).
