# Supplementary material for: Efficiency optimization for large-scale droplet-based electricity generator arrays with integrated microsupercapacitor arrays
Source: Nat Commun. 2025 Sep 26;16:8530. doi: 10.1038/s41467-025-64289-y (PMC12475142; doi:10.1038/s41467-025-64289-y)
Supplement: Supplementary file 3 — Description of Additional Supplementary Files [file 41467_2025_64289_MOESM3_ESM.pdf]

Description of Additional Supplementary Files

Supplementary Video 1

Description: LED lighted up by 200-cell MSC arrays (Charged 30 s by 30-cell DEG arrays).

Supplementary Video 2

Description: LED lighted up by 400-cell MSC arrays (Charged 30 s by 30-cell DEG arrays).

Supplementary Video 3

Description: Sensor Powered by 200-cell MSC arrays (Charged 30 s by 30-cell DEG arrays).

Supplementary Video 4

Description: LED lighted up by 200-cell MSC arrays (Charged 30 s by 30-cell DEG arrays with rain water) Supplementary Video 5: Calculator Powered by 200-cell MSC arrays (Charged 30 s by 30-cell DEG arrays with rain water).
